# Foetal growth, birth transition, enteral nutrition and brain light scattering

**DOI:** 10.1038/s41598-021-00624-9

**Published:** 2021-10-29

**Authors:** Osuke Iwata, Sachiko Iwata, Tsuyoshi Kurata, Kennosuke Tsuda, Koya Kawase, Masahiro Kinoshita, Yung-Chieh Lin, Mamoru Saikusa, Yuko Araki, Sachio Takashima, Motoki Oda, Etsuko Ohmae, Shiji Saitoh

**Affiliations:** 1 Center for Human Development and Family Science, Department of Pediatrics and Neonatology, Nagoya City University Graduate School of Medical Sciences, Nagoya City, Aichi 467-8601 Japan; 2 Centre for Developmental and Cognitive Neuroscience, Department of Paediatrics and Child Health, Kurume University School of Medicine, Kurume City, Fukuoka Japan; 3 Department of Pediatrics, National Cheng Kung University Hospital, College of Medicine, National Cheng-Kung University, Tainan City, Taiwan; 4 Faculty of Informatics, Shizuoka University, Hamamatsu City, Shizuoka Japan; 5 Yanagawa Institute for Developmental Disabilities, International University of Health and Welfare, Yanagawa City, Fukuoka Japan; 6 Central Research Laboratory, Hamamatsu Photonics K.K., Hamamatsu City, Shizuoka Japan

**Keywords:** Developmental biology, Neuroscience, Biomarkers, Diseases, Medical research, Neurology, Optics and photonics

## Abstract

If the brain structure is assessed at neonatal intensive care units, covert clinical events related with subtle brain injury might be identified. The reduced scattering coefficient of near-infrared light (μ_S_’) obtained using time-resolved near-infrared spectroscopy from the forehead of infants is associated with gestational age, body weight and Apgar scores, presumably reflecting subtle changes of the brain related to foetal growth and birth transition. One hundred twenty-eight preterm and term infants were studied to test whether μ_S_’ obtained from the head at term-equivalent age is associated with foetal growth, birth transition and nutritional status after birth, which are key independent variables of developmental outcomes. As potential independent variables of μ_S_’, birth weight, Apgar scores, age at full enteral feeding and post-conceptional age at the study were assessed to represent foetal growth, birth transition and nutritional status after birth. Subsequently, higher μ_S_’ values were associated with higher Apgar scores (*p* = 0.003) and earlier establishment of enteral feeding (*p* < 0.001). The scattering property of near-infrared light within the neonatal brain might reflect changes associated with birth transition and nutritional status thereafter, which might be used as a non-invasive biomarker to identify covert independent variables of brain injury in preterm infants.

## Introduction

Advances in neonatal intensive care have significantly improved the survival rate of preterm infants^1,2^. However, a considerable fraction of extremely preterm infants develop cognitive impairments even in the absence of major cerebral lesions, such as intracranial haemorrhage and periventricular leukomalacia^3,4^. Magnetic resonance imaging (MRI) studies in preterm infants have demonstrated the relationship between subtle brain lesions at term equivalent age and long-term cognitive impairments^5–7^. However, because of the cost, time and safety associated with the scan, MRI is usually performed only once before discharge from the hospital, causing difficulty in identification of the upstream events associated with subtle brain lesions. Reliable tools for the assessment of subtle change of the brain structure, which can be assessed before and after clinical events at the cot-side, may help distinguish the upstream events responsible for subtle cerebral lesions and cognitive impairments in preterm infants.

Near-infrared spectroscopy (NIRS) is a handy, non-invasive tool, which has been used to analyse the tissue oxygen metabolism in the brains of newborn infants^8–11^. Near-infrared light penetrates the intact scalp, skull and cerebral tissue more efficiently than visible light, and is mainly absorbed by blood haemoglobin, the level of which depends on the binding of haemoglobin to oxygen^12^. Thus, fractions of oxygenated and deoxygenated haemoglobin are calculated using light absorption coefficient (μ_a_) obtained from the near-infrared light of different wavelengths^13^. Time-resolved near-infrared spectroscopy (TR-NIRS) is a relatively new technique, which enables simultaneous quantification of μ_a_ and reduced scattering coefficient (μ_S_’)^14,15^. Unlike μ_a_ predominantly provides information regarding tissue oxygenation, μ_S_’ is an index of light scattering, which is theoretically determined by the structural complexity of tissue^13^. When preterm infants were studied shortly after birth, μ_S_’ values obtained from the forehead showed a positive linear correlation with gestational age^16^. Our study in preterm and term infants further confirmed that μ_S_’ values obtained shortly after birth were associated with variables, such as antenatal glucocorticoid, emergency delivery, gestational age, body size, Apgar scores, requirement for mechanical ventilation and blood gas data at birth, suggesting the possibility that μ_S_’ might reflect subtle structural changes in the brain associated with antenatal growth, peripartum stress and birth transition^17^. However, little is known regarding the relationship between μ_S_’ values obtained from the head of newborn infants and their downstream clinical outcomes.

The aim of this study was to test the association of μ_S_’ measured at term-equivalent period with intrauterine growth, birth transition and nutrition after birth, which are short-term surrogate markers for neurodevelopmental outcomes of hospitalised newborn infants^18–21^.

## Results

**Figure 1:**
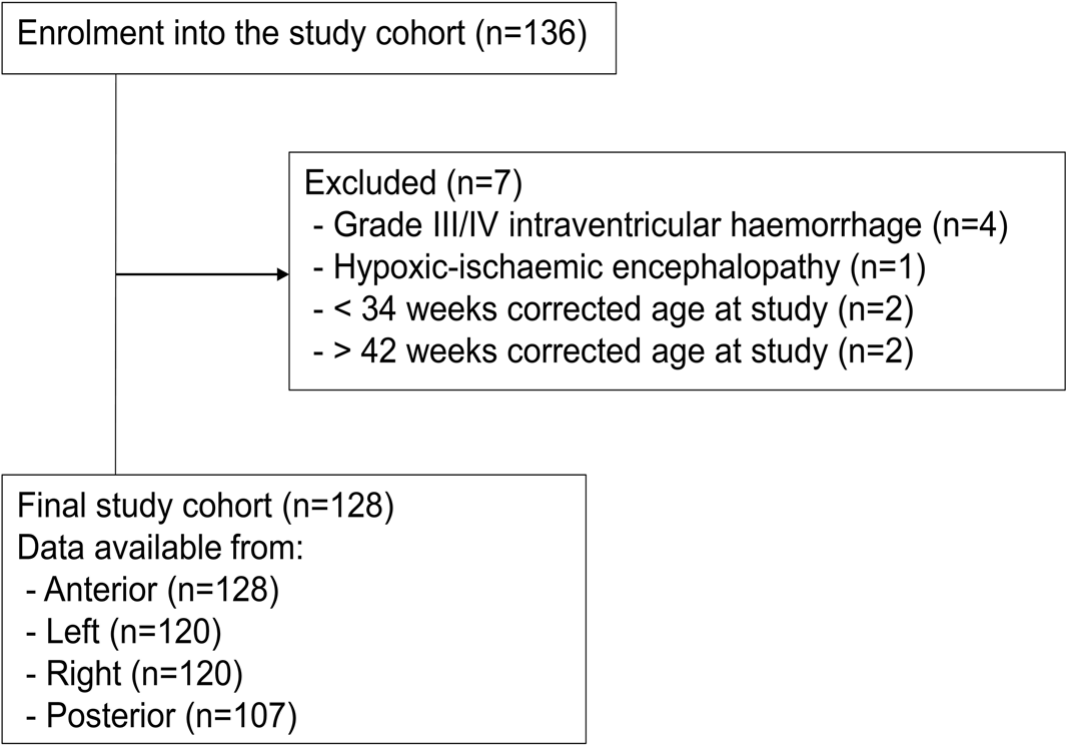
Profile of the study population. A diagram depicting the flow of the study population.

Four infants, who developed grade III/IV intraventricular haemorrhage, and one infant, who developed hypoxic-ischaemic encephalopathy, were excluded, leaving 128 infants within the final study cohort (Fig. 1). These infants had a gestation period of 32.0 ± 4.2 weeks and weighed 1564 ± 688 g at birth, and were studied on 44.8 ± 28.3 days of age or 38.6 ± 2.1 weeks post-conceptional age (Table 1).

**Table 1:** Background characteristics of 128 infants within the study cohort.

**Maternal and antenatal variables**	
Antenatal glucocorticoid	61 (47.7%)
Multiple pregnancy	39 (30.5%)
Emergency caesarean delivery	55 (43.0%)
**Variables at birth**	
Gestational age (week)	32.0 ± 4.2
Body weight at birth (g)	1564 ± 688
Z-score of above	− 0.9 ± 1.3
Head circumference at birth (cm)	28.3 ± 3.5
Z-score of above	− 0.2 ± 1.1
Male sex	68 (53.1%)
Cord blood pH	7.299 ± 0.146
Apgar score (1 min)	7 (4, 8)
Apgar score (5 min)	8 (7, 9)
Hypoglycaemia < 48 h of birth	8 (6.3%)
**Variables during hospital stay**	
Indomethacin for patent ductus arteriosus	38 (29.7%)
Surgical closure of patent ductus arteriosus	1 (0.8%)
Grade I/II intraventricular haemorrhage	6 (4.7%)
Periventricular leukomalacia	1 (0.8%)
Full enteral feeding ≥ 100 mL/kg/d (day)	7.7 ± 5.3
Days on invasive ventilation	10.3 ± 18.4
Chronic lung disease^a^	22 (17.2%)
**Variables at study**	
Post-conceptional age (week)	38.6 ± 2.1
Postnatal age (day)	44.8 ± 28.3
Body weight (g)	2775 ± 408
Blood haemoglobin (g/dL)	12.6 ± 2.4
µ_a_ (cm^−1^)	0.126 ± 0.025
μ_S_’ (cm^−1^)	6.453 ± 1.416

Values are number (%), mean ± standard deviation or median (lower/upper quartiles).

*µ*_*a*_ absorption coeffieicnt, *µ*_*s*_’ reduced scattering coefficient.

^a^Assessed at 36 weeks post-conceptional age (or on day 28 for those born later than 32 weeks gestation).

For the left and right temporal regions and the posterior region, data were not obtained for 8, 8 and 21 infants, respectively, because of insufficient signals from the head (n = 15), poor probe contact (n = 4) and the use of a cap device for non-invasive respiratory support (n = 2). No further data were excluded because of their poor quality or reproducibility. The mean μ_a_ and μ_S_’ values for all wavelengths and head positions were 0.126 ± 0.025 cm^−1^ and 6.453 ± 1.416 cm^−1^, respectively.

### Dependence of μ_a_ and μ_S_’ on wavelengths and head positions

The wavelength of 836 nm was associated with higher μ_a_ values, whereas the wavelength of 791 nm was associated with lower μ_a_ values compared to those of 761 nm (both *p* < 0.001) (Table 2). The right temporal and posterior regions of the head were associated with higher μ_a_ values compared to those of the anterior region (both *p* < 0.001).

**Table 2:** Dependence of µ_a_ and μ_S_’ on clinical variables: univariate analysis.

Independent variables	Correlation with µ_a_·10^2^				Correlation with μ_S_’			
	B	95% CI		*p*	B	95% CI		*p*
		Lower	Upper			Lower	Upper	
**Wavelength (vs. 761 nm)**^**†**^								
836 nm	0.663	0.530	0.797	< 0.001	− 0.337	− 0.375	− 0.300	< 0.001
791 nm	− 1.508	− 1.634	− 1.382	< 0.001	− 0.169	− 0.227	− 0.112	< 0.001
Position (vs. anterior)**								
Posterior	3.140	2.809	3.471	< 0.001	1.414	1.129	1.699	< 0.001
Right	0.449	0.199	0.699	< 0.001	1.554	1.302	1.807	< 0.001
Left	0.196	− 0.015	0.406	0.068	0.909	0.667	1.152	< 0.001
Maternal and antenatal variables**^†^								
Male sex	− 0.530	− 1.144	0.083	0.090	− 0.210	− 0.528	0.107	0.195
Multiple pregnancy	− 0.656	− 1.190	− 0.121	0.016	− 0.241	− 0.627	0.144	0.220
Antenatal glucocorticoid	− 1.274	− 1.829	− 0.719	< 0.001	− 0.392	− 0.702	− 0.083	0.013
Hypoglycaemia < 48 h of birth	0.746	− 1.385	2.878	0.492	0.649	− 0.233	1.531	0.149
Variables at birth**^†^								
Indomethacin for patent ductus arteriosus	− 0.481	− 1.084	0.122	0.118	− 0.519	− 0.810	− 0.228	< 0.001
Emergency caesarean delivery	− 0.468	− 1.049	0.113	0.114	− 0.296	− 0.603	0.010	0.058
Chronic lung disease*	− 0.484	− 1.059	0.092	0.099	− 0.345	− 0.711	0.022	0.065
Intraventricular haemorrhage	− 0.254	− 0.742	0.235	0.309	0.302	− 0.396	1.000	0.396
Gestational age (week)	0.167	0.066	0.268	0.001	0.074	0.035	0.112	< 0.001
Body weight (kg)	0.124	0.072	0.176	< 0.001	0.034	0.007	0.060	0.012
Z-score of above	0.129	− 0.076	0.334	0.218	− 0.041	− 0.157	0.074	0.484
Head circumference (cm)	0.142	0.043	0.240	0.005	0.060	0.013	0.106	0.012
Z-score of above	− 0.039	− 0.382	0.305	0.824	− 0.140	− 0.280	0.000	0.050
Cord blood pH per 0.1 change	− 0.280	− 0.466	− 0.093	0.003	− 0.054	− 0.142	0.035	0.233
Apgar score (1 min)	0.009	− 0.128	0.146	0.899	0.064	0.003	0.125	0.039
Apgar score (5 min)	− 0.097	− 0.314	0.120	0.382	0.088	0.009	0.168	0.029
Variables at study**^†^								
Postnatal age (day)	− 0.021	− 0.033	− 0.008	0.001	− 0.008	− 0.014	− 0.003	0.003
Post-conceptional age (week)	0.131	− 0.048	0.310	0.151	0.066	− 0.013	0.145	0.104
Body weight (kg)	0.065	0.004	0.126	0.036	− 0.006	− 0.044	0.032	0.753
Blood haemoglobin (g/dL)	0.568	0.462	0.675	< 0.001	0.057	− 0.018	0.133	0.135
Full enteral feeding ≥ 100 mL/kg/d (day)	− 0.027	− 0.077	0.022	0.278	− 0.052	− 0.077	− 0.027	< 0.001
μ_S_’ (cm^−1^)	0.573	0.314	0.833	< 0.001	Not applicable			
µ_a_ (cm^−1^)	Not applicable				22.521	16.167	28.875	< 0.001

*B* regression coefficient, *CI* confidence interval, *µ*_*a*_ absorption coefficient, *µ*_*s*_’ reduced scattering coefficient.

*Assessed at 36 weeks post-conceptional age (or on day 28 for those born later than 32 weeks gestation).

Findings are adjusted for the wavelengths of near-infrared light** and position of the head^†^.

The wavelengths of 791 and 836 nm were associated with lower μ_S_’ values compared to those of 761 nm (both *p* < 0.001). The left and right temporal and posterior regions of the head were associated with higher μ_S_’ values compared to the anterior region (all *p* < 0.001).

### Dependence of μ_a_ and μ_S_’ on clinical variables: univariate analysis

**Figure 2:**
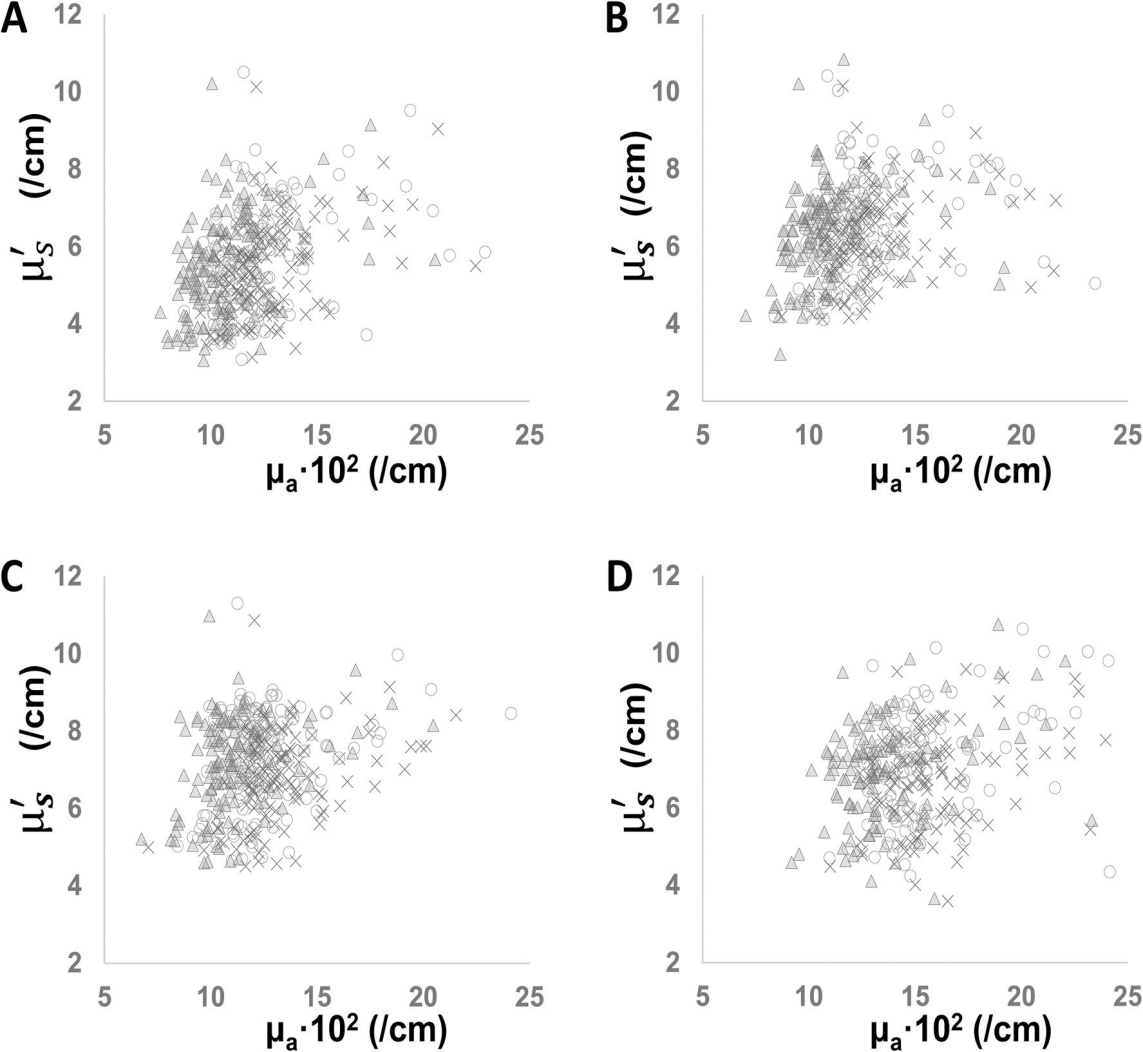
Dependence of μ_S_’ on µ_a_ in four head regions. Scatter plots demonstrating relationships between µ_a_ and μ_S_’ obtained from the anterior (**A**), left-temporal (**B**), right-temporal (**C**) and posterior (**D**) head regions for wavelengths of 761 nm (circle), 791 nm (triangle) and 836 nm (cross). µ_a_, absorption coeffieicnt. μ_S_’, reduced scattering coefficient.

The higher μ_a_ values were positively associated with gestational age (*p* = 0.001), body weight at birth (*p* < 0.001), blood haemoglobin level at study (*p* < 0.001) and μ_S_’ values (*p* < 0.001), and negatively associated with antenatal glucocorticoid (*p* < 0.001), cord blood pH (*p* = 0.003) and postnatal age at study (*p* = 0.001); relationships with multiple pregnancy (*p* = 0.016), head circumference at birth (*p* = 0.005) and body weight at study (*p* = 0.036) were lost after correction for multiple comparisons (all adjusted for the wavelengths and head positions; Table 2 and Fig. 2).

The μ_S_’ level was positively associated with gestational age (*p* < 0.001) and μ_a_ values (*p* < 0.001), and negatively associated with indomethacin for patent ductus arteriosus (*p* < 0.001) and postnatal age to achieve full enteral feeding (*p* < 0.001); relationships with antenatal glucocorticoid (*p* = 0.013), body weight and head circumference at birth (both *p* = 0.012), Apgar scores at 1 and 5 min (*p* = 0.039 and 0.029, respectively) and postnatal age at study (*p* = 0.003) were lost after correction for multiple comparisons (all adjusted for the wavelengths and head positions; Table 2). See Online Supplemental Tables S1–S3 for findings from analyses performed for each wavelength.

### Dependence of μ_S_’ and μ_a_ on clinical variables: multivariate analysis

Higher μ_a_ values were associated with greater age to achieve full enteral feeding (*p* = 0.049), greater post-conceptional age at study (*p* = 0.015), higher blood haemoglobin levels at study (*p* < 0.001) and higher μ_S_’ values (*p* < 0.001) (Table 3). Higher μ_S_’ values were associated with higher Apgar scores at 5 min (*p* = 0.003), smaller age to achieve full enteral feeding (*p* < 0.001) and higher μ_a_ values (*p* < 0.001). See Online Supplemental Tables S4–S6 for findings from analyses performed for each wavelength.

**Table 3:** Dependence of µ_a_ and μ_S_’ on clinical variables: multivariate analysis.

	Correlation with µ_a_·10^2^				Correlation with μ_S_’			
	B	95% CI		*P*	B	95% CI		*p*
		Lower	Upper			Lower	Upper	
**Independent variables**								
Body weight at birth (per 100 g)	− 0.015	− 0.053	0.022	0.420	− 0.021	− 0.049	0.007	0.136
Apgar score (5 min)	− 0.011	− 0.165	0.143	0.890	0.096	0.033	0.159	0.003
Full enteral feeding ≥ 100 mL/kg/d (day)	0.027	0.000	0.053	0.049	− 0.051	− 0.077	− 0.026	< 0.001
Post-conceptional age at study (week)	0.141	0.028	0.255	0.015	0.055	− 0.009	0.119	0.093
**Covariates**								
Antenatal glucocorticoid	− 0.156	− 0.525	0.214	0.409	− 0.035	− 0.335	0.265	0.820
Multiple pregnancy	− 0.254	− 0.587	0.079	0.135	− 0.153	− 0.486	0.180	0.369
Male sex	− 0.080	− 0.436	0.277	0.662	− 0.225	− 0.496	0.046	0.103
Blood haemoglobin (g/dL)	0.569	0.466	0.671	< 0.001	Not involved			
μ_S_’ (cm^−1^)	0.450	0.303	0.596	< 0.001	Not applicable			
µ_a_ (cm^−1^)	Not applicable				22.692	15.952	29.432	< 0.001

The model is also adjusted for the wavelengths of near-infrared light and position of the head.

*B* regression coefficient, *CI* confidence interval, *µ*_*a*_ absorption coefficient, *µ*_*s*_’ reduced scattering coefficient.

## Discussion

Building on previous studies of TR-NIRS, which suggested that the light scattering within the brain shortly after birth is dependent on variables related to foetal growth, antenatal stress and birth transition, we have demonstrated that higher μ_S_’ values obtained at term-equivalent age were associated with higher Apgar scores and earlier establishment of enteral nutrition. μ_S_’ ﻿can be a unique and clinically useful biomarker of subtle changes in the brains of newborn infants with respect to antenatal stress, birth transition and nutritional status after birth.

Light scattering within a tissue theoretically increases with relatively more complex microstructures due to increased reflection and path length of near-infrared light^14^. Thus μ_S_’ has a potential to provide microstructural information of the brain. Ijichi and colleagues first reported that μ_S_’ values of near-infrared light obtained shortly after birth from the foreheads of newborn infants with a gestation age of 30–41 weeks depended on gestational age^16^. Our previous study confirmed that μ_S_’ values obtained from the foreheads of preterm and term infants assessed shortly after birth were dependent on body size and Apgar scores, as well as on gestational age. These findings suggest the possible utility of μ_S_’ values as a non-invasive marker to evaluate subtle differences in the brain subsequent to foetal maturation, antenatal stress and birth transition^17^. Our current study further verified that the μ_S_’ value obtained at term equivalent period is associated with both clinical variables at birth and those related to the nutritional status of the infant after birth. Intrauterine growth and maturation, intrapartum stress and response and postpartum nutrition and growth constitute key independent variables of the neurodevelopmental outcomes of the infant^18–21^. If the consequence of the intrinsic maturity, extrinsic stress, birth transition and nutritional status of the infant can be assessed using μ_S_’ values obtained from the heads of newborn infants, along with other substantiations, μ_S_’ might serve as a clinically useful biomarker of cerebral maturation and damage. Future studies need to address the contribution of other potential independent variables of light scattering as measured from the scalp, such as the gyration of the brain and developmental changes in the layer of cerebrospinal fluid.

With regard to the absorption of near-infrared light, only modest relationships were observed between higher μ_a_ values and longer time to achieve full enteral feeding and greater post-conceptional age at the time of the study; robust correlations were only observed between μ_a_ values and priori covariates of the wavelengths of light, head position and blood haemoglobin concentration at the time of the study. Given that absorption of near-infrared light within the range of 750–850 nm is primarily determined by the tissue haemoglobin concentrations^14,15^, μ_a_ values might reflect the maturation of the cerebral tissue via increased complexity of the cerebral vessels and subsequent blood volume. Progression of anaemia and increase in the cerebral blood flow and volume with increasing postnatal age might also affect the dependence of μ_a_ values on clinical variables^12^.

### Strengths and limitations

We were able to elucidate the clinical variables potentially determining the property of light absorption and scattering within the brain in a relatively large cohort of newborn infants. However, we were unable to present a direct association between μ_S_’ values and microstructure of the brain. As described in the previous section, the observed relationships between μ_S_’, μ_a_ and clinical variables can be affected by a range of clinical biases. For example, extremely preterm infants are relatively anaemic at birth and the anaemia progresses with postnatal age without transfusion, potentially leading to lower blood haemoglobin and μ_a_ levels with greater gestational age at birth and greater postnatal age at the time of TR-NIRS study. Although we carefully selected independent variables and covariates to minimise the bias, the findings might still be affected by the bias derived from the collinearity between the variables. Our study cohort comprised newborn infants, who were hospitalised at a tertiary neonatal intensive care unit. Although the observed μ_a_ and μ_S_’ values were comparable to those reported in healthy newborn infants^22^, extrapolation of our findings into physiological transition and growth in healthy newborn infants must be done cautiously. Finally, the longitudinal follow-up study of the study population is still underway, resulting in the lack of outcome information in association with the light absorption and scattering properties.

## Conclusions

The μ_S_’ values of the near-infrared light obtained at term-equivalent period from the heads of newborn infants were associated with Apgar scores and postnatal age when full enteral feeding was achieved, suggesting a correlation between the light scattering property and stress-response at birth and nutritional status of the infant thereafter. With further validations, μ_S_’ might serve as a biomarker to distinguish the variation of the microstructural complexity of the brain tissue subsequent to different maturational stage, antenatal stress, tissue damage and repair, nutritional status and growth. Associations between the μ_S_’ values and detailed clinical courses, macro- and microstructural MRI findings and neuro-developmental outcomes need to be addressed to assess the clinical utility of this non-invasive cot-side tool.

## Materials and methods

This study was conducted in compliance with the Declaration of Helsinki under the approval of the Ethics Committee of Kurume University School of Medicine (reference number: 12128). Informed parental consent was obtained for each participating newborn infant. All methods were carried out in accordance with relevant guidelines and regulations.

### Study population

This study was performed as a secondary analysis of a prospective longitudinal study, which was performed between June 2009 and January 2015 to serially collected the TR-NIRS data of preterm and term infants hospitalised at a tertiary neonatal intensive care centre of Kurume University Hospital (Kurume, Fukuoka, Japan). Independent variables of μ_S_’ values obtained shortly after birth from a part (n = 60) of the current cohort have been reported in a previous study^17^. Of 136 newborn infants within the original study cohort, 132 infants, who had TR-NIRS data obtained between 34 and 42 weeks postconceptional age, were considered. Infants with chromosomal aberration, malformation syndrome, grade III/IV intraventricular haemorrhage, hypoxic-ischaemic encephalopathy, congenital hydrocephalus and other major cerebral anomalies were excluded.

### Data collection

The μ_a_ and μ_S_’ values were obtained from the heads of the infants for three wavelengths, 761, 791 and 836 nm, using a TR-NIRS system (TRS-10, Hamamatsu Photonics K.K., Hamamatsu, Shizuoka, Japan)^17^. This system employs the time-correlated single photon counting method to create time response profiles of pulsed laser light penetrating an object. The time response profiles were then fitted into a photon diffusion equation using the nonlinear least square fitting method to obtain μ_a_ and μ_S_’ for each wavelength^14^. Although the data acquisition for the original study was repeated with intervals of approximately 1 week from birth to discharge, for the current study, a particular value obtained between 34 and 42 weeks of post-conceptional age (closest to 40 weeks gestation if there were multiple records) was used to represent each infant.

Data were acquired when the infant was clinically stable and asleep or calmly awake. The TR-NIRS probes were inserted into a rubber holder, with an inter-optode distance of 3 cm, and was applied to a relatively flat part of the head. Data acquisition (10 s) was repeated five times for each of the frontal, left and right temporo-parietal and occipital regions by repositioning the probe each time. In our previous study, which acquired TR-NIRS data using the same protocol to the current one^17^, standard deviations of μ_a_ and μ_S_’ values for five successionally obtained data within the same head position and infant were, in average, 2.4% and 2.7%, respectively. Based on these small intra-individual and intra-regional differences in μ_a_ and μ_S_’ values, five readings each of μ_a_ and μ_S_’ were averaged for each brain region. We confirmed the degree of fit to the photon diffusion equation using the conversion chi-square value index of between 0.8 and 1.2^23^. Data were not collected for brain regions with poor probe contact (typically due to the lack of flat surfaces or use of cap devices for non-invasive respiratory support), poor fit to the photon diffusion equation or insufficient signal-to-noise ratio with the count rate < 100 K counts/s or relative dark- to peak-count ratio of > 0.1. The data were retrospectively assessed to identify those with poor quality or intra-regional reproducibility before being processed for further analysis.

### Clinical information

The clinical background information was obtained from the electronic records of the patients, including (1) maternal and antenatal variables (antenatal glucocorticoid, multiple pregnancies and emergency caesarean delivery), (2) variables at and shortly after birth (sex, cord blood pH, Apgar scores at 1 min and 5 min, gestational age, body weight, head circumference, hypoglycaemia within 48 h of birth, indomethacin for the treatment of the patent ductus arteriosus, grade I/II intraventricular haemorrhage and periventricular leukomalacia, (3) variables associated with clinical variables of infants after the transitional period (body weight on the day of study, age when full enteral feeding of > 100 ml/kg/d was achieved and chronic lung disease assessed 36 weeks post-conceptional age or on day 28, whichever was later). In order to assess the influence of intrauterine growth on μ_S_’ values, body weight and head circumference at birth were expressed as z-scores in accordance with the New Japanese Neonatal Anthropometric Charts for Gestational Age at Birth^24^.

### Data analysis

To minimise biases owing to missing data, multiple imputation of the missing values of less than 10% (excluding for μ_a_ and μ_S_’) was performed (n = 5 imputations), based on the correlation between variables with missing values and other characteristics of the participants (SPSS ver. 22.0, IBM, Armonk, NY, U.S.A.). Although the property of μ_a_ was out of our study scope, independent variables of both μ_a_ and μ_S_’ were assessed to clarify the possible influence of light absorption to the relationship between μ_S_’ values and clinical variables. The generalised estimating equation with a linear model was used to account for repeated sampling of TR-NIRS data for three near-infrared light wavelengths and four head regions. Although the influence of the wavelength is much greater on μ_a_ than on μ_S_’^16,17^, the three wavelengths were incorporated within the model for consistency in the analytical procedure. Crude effects of clinical variables on μ_a_ and μ_S_’ values were assessed using the univariate model adjusting for the wavelengths and head positions. *p* values < 0.002 were assumed to be significant, correcting multiple comparisons of 25 variables. The final models to explain μ_a_ and μ_S_’ values were developed based on our hypothesis, which employed the body weight at birth, Apgar scores at 5 min, age to achieve full enteral feeding and post-conceptional age at study; the model was also adjusted for priori covariates, which were known independent variables of clinical outcomes (antenatal glucocorticoid, multiple pregnancies and sex), μ_a_ (wavelength, position of the head and μ_S_’) and μ_S_’ (wavelength, position of the head and μ_a_). Data were presented as mean ± standard deviation unless specified otherwise.

## Supplementary Information

Supplementary Tables.
