# Supplementary material for: Foetal growth, birth transition, enteral nutrition and brain light scattering
Source: Sci Rep. 2021 Oct 29;11:21318. doi: 10.1038/s41598-021-00624-9 (PMC8556386; doi:10.1038/s41598-021-00624-9)
Supplement: Supplementary file 1 — Supplementary Tables. [file 41598_2021_624_MOESM1_ESM.docx]

**Title:**

**Foetal growth, birth transition, enteral nutrition and brain light scattering**

**Authors:**

Osuke Iwata^1,2^; Sachiko Iwata^1^; Tsuyoshi Kurata^2^; Kennosuke Tsuda^1^; Koya Kawase^1^; Masahiro Kinoshita^2^; Yung-Chieh Lin^3^; Mamoru Saikusa^2^; Yuko Araki^4^; Sachio Takashima^5^; Motoki Oda^6^; Etsuko Ohmae^6^ and Shiji Saitoh^1^.

**Affiliations:**

^1^ Center for Human Development and Family Science, Department of Pediatrics and Neonatology, Nagoya City University Graduate School of Medical Sciences, Nagoya City, Aichi, Japan.

^2^ Centre for Developmental and Cognitive Neuroscience, Department of Paediatrics and Child Health, Kurume University School of Medicine, Kurume City, Fukuoka, Japan.

^3^ Department of Pediatrics, National Cheng Kung University Hospital, College of Medicine, National Cheng-Kung University, Tainan City, Taiwan.

^4^ Faculty of Informatics, Shizuoka University, Shizuoka City, Shizuoka, Japan.

^5^ Yanagawa Institute for Developmental Disabilities, International University of Health and Welfare, Yanagawa City, Fukuoka, Japan.

^6^ Central Research Laboratory, Hamamatsu Photonics K.K., Hamamatsu City, Shizuoka, Japan.

Online supplemental table 1: Dependence of µ_a_ and µ’_s_ obtained with the wavelength of 761nm on clinical variables: univariate analysis

|  | **Correlation with µ_a_·10^2^** | | | | | **Correlation with** $\boldsymbol{\mu}_{\boldsymbol{s}}^{\boldsymbol{'}}$ | | | |
| --- | --- | --- | --- | --- | --- | --- | --- | --- | --- |
| **Independent variables** | **B** | **95% CI** | | **P** |  | **B** | **95% CI** | | **P** |
|  |  | **Lower** | **Upper** |  |  |  | **Lower** | **Upper** |  |
| Position (vs. anterior) | | |  |  |  |  |  |  |  |
| Posterior | 3.193 | 2.842 | 3.543 | <0.001 |  | 1.510 | 1.211 | 1.810 | <0.001 |
| Right | 0.350 | 0.080 | 0.615 | 0.009 |  | 1.645 | 1.380 | 1.909 | <0.001 |
| Left | 0.120 | -0.097 | 0.336 | 0.266 |  | 0.956 | 0.702 | 1.211 | <0.001 |
| Maternal and antenatal variables* | | |  |  |  |  |  |  |  |
| Male sex | -0.527 | -1.164 | 0.111 | 0.105 |  | -0.216 | -0.546 | 0.115 | 0.201 |
| Multiple pregnancy | -0.628 | -1.194 | -0.062 | 0.030 |  | -0.254 | -0.656 | 0.147 | 0.214 |
| Antenatal glucocorticoid | -1.308 | -1.886 | -0.729 | <0.001 |  | -0.466 | -0.785 | -0.146 | 0.004 |
| Hypoglycaemia <48h of birth | 0.552 | -1.603 | 2.707 | 0.615 |  | 0.669 | -0.276 | 1.615 | 0.165 |
| Variables at birth* | |  |  |  |  |  |  |  |  |
| Indomethacin for patent ductus arteriosus | -0.238 | -0.922 | 0.445 | 0.494 |  | -0.537 | -0.836 | -0.238 | <0.001 |
| Emergency caesarean delivery | -0.433 | -1.036 | 0.170 | 0.160 |  | -0.306 | -0.626 | 0.014 | 0.061 |
| Chronic lung disease** | -0.318 | -0.937 | 0.301 | 0.313 |  | -0.353 | -0.735 | 0.030 | 0.071 |
| Intraventricular haemorrhage | -0.164 | -0.794 | 0.465 | 0.609 |  | 0.336 | -0.404 | 1.075 | 0.373 |
| Gestational age (week) | 0.138 | 0.027 | 0.249 | 0.015 |  | 0.082 | 0.043 | 0.121 | <0.001 |
| Body weight (kg) | 0.118 | 0.062 | 0.173 | <0.001 |  | 0.042 | 0.017 | 0.067 | 0.001 |
| Z-score of above | 0.194 | -0.043 | 0.431 | 0.109 |  | -0.022 | -0.143 | 0.099 | 0.717 |
| Head circumference (cm) | 0.114 | -0.008 | 0.236 | 0.066 |  | 0.069 | 0.022 | 0.116 | 0.004 |
| Z-score of above | 0.041 | -0.335 | 0.418 | 0.829 |  | -0.120 | -0.269 | 0.029 | 0.114 |
| Cord blood pH per 0.1 change | -0.300 | -0.493 | -0.106 | 0.002 |  | -0.072 | -0.171 | 0.028 | 0.157 |
| Apgar score (1 min) | -0.034 | -0.181 | 0.114 | 0.653 |  | 0.061 | -0.004 | 0.125 | 0.065 |
| Apgar score (5 min) | -0.158 | -0.402 | 0.087 | 0.206 |  | 0.084 | 0.000 | 0.168 | 0.051 |
| Variables at study* | |  |  |  |  |  |  |  |  |
| Postnatal age (day) | -0.016 | -0.029 | -0.003 | 0.018 |  | -0.009 | -0.015 | -0.003 | 0.002 |
| Post-conceptional age (week) | 0.138 | -0.046 | 0.322 | 0.141 |  | 0.082 | 0.000 | 0.165 | 0.051 |
| Body weight (kg) | 0.093 | 0.020 | 0.166 | 0.012 |  | 0.005 | -0.035 | 0.045 | 0.813 |
| Blood haemoglobin (g/dL) | 0.585 | 0.476 | 0.695 | <0.001 |  | 0.081 | 0.005 | 0.156 | 0.036 |
| Full enteral feeding ≥100mL/Kg/d (day) | 0.000 | -0.057 | 0.058 | 0.987 |  | -0.053 | -0.080 | -0.027 | <0.001 |
| $\boldsymbol{\mu}_{\boldsymbol{s}}^{\boldsymbol{'}}$(cm^-1^) | 0.502 | 0.251 | 0.753 | <0.001 |  | Not applicable | | | |
| µ_a_ (cm^-1^) | Not applicable | | | |  | 20.296 | 12.222 | 28.371 | <0.001 |

*Findings are adjusted for the position of the head.

**Assessed at 36 weeks post-conceptional age (or on day 28 for those born later than 32 weeks gestation).

Abbreviations: B, regression coefficient. CI, confidence interval. µ_a_, absorption coeffieicnt. $\boldsymbol{\mu}_{\boldsymbol{s}}^{\boldsymbol{'}}$, reduced scattering coefficient.

Online supplemental table 2: Dependence of µ_a_ and µ’_s_ obtained with the wavelength of 791nm on clinical variables: univariate analysis

|  | **Correlation with µ_a_·10^2^** | | | | | **Correlation with** $\boldsymbol{\mu}_{\boldsymbol{s}}^{\boldsymbol{'}}$ | | | |
| --- | --- | --- | --- | --- | --- | --- | --- | --- | --- |
| **Independent variables** | **B** | **95% CI** | | **P** |  | **B** | **95% CI** | | **P** |
|  |  | **Lower** | **Upper** |  |  |  | **Lower** | **Upper** |  |
| Position (vs. anterior) | | |  |  |  |  |  |  |  |
| Posterior | 3.028 | 2.710 | 3.346 | <0.001 |  | 1.420 | 1.131 | 1.709 | <0.001 |
| Right | 0.443 | 0.206 | 0.680 | <0.001 |  | 1.558 | 1.303 | 1.814 | <0.001 |
| Left | 0.209 | 0.007 | 0.411 | 0.040 |  | 0.923 | 0.678 | 1.169 | <0.001 |
| Maternal and antenatal variables* | | |  |  |  |  |  |  |  |
| Male sex | -0.576 | -1.167 | 0.015 | 0.056 |  | -0.249 | -0.584 | 0.085 | 0.144 |
| Multiple pregnancy | -0.576 | -1.096 | -0.055 | 0.030 |  | -0.214 | -0.610 | 0.182 | 0.290 |
| Antenatal glucocorticoid | -1.184 | -1.722 | -0.645 | <0.001 |  | -0.384 | -0.711 | -0.058 | 0.021 |
| Hypoglycaemia <48 h of birth | 0.742 | -1.263 | 2.747 | 0.468 |  | 0.646 | -0.224 | 1.516 | 0.145 |
| Variables at birth* | |  |  |  |  |  |  |  |  |
| Indomethacin for patent ductus arteriosus | -0.446 | -1.029 | 0.137 | 0.134 |  | -0.487 | -0.795 | -0.179 | 0.002 |
| Emergency caesarean delivery | -0.359 | -0.922 | 0.204 | 0.212 |  | -0.222 | -0.549 | 0.105 | 0.183 |
| Chronic lung disease** | -0.426 | -1.006 | 0.154 | 0.150 |  | -0.320 | -0.708 | 0.067 | 0.105 |
| Intraventricular haemorrhage | -0.117 | -0.661 | 0.428 | 0.674 |  | 0.373 | -0.336 | 1.081 | 0.303 |
| Gestational age (week) | 0.148 | 0.047 | 0.249 | 0.004 |  | 0.066 | 0.020 | 0.112 | 0.005 |
| Body weight (kg) | 0.098 | 0.032 | 0.164 | 0.004 |  | 0.023 | -0.017 | 0.063 | 0.267 |
| Z-score of above | 0.041 | -0.159 | 0.242 | 0.685 |  | -0.083 | -0.233 | 0.067 | 0.279 |
| Head circumference (cm) | 0.118 | 0.022 | 0.214 | 0.016 |  | 0.050 | -0.008 | 0.108 | 0.090 |
| Z-score of above | -0.125 | -0.445 | 0.195 | 0.443 |  | -0.178 | -0.347 | -0.010 | 0.038 |
| Cord blood pH per 0.1 change | -0.257 | -0.442 | -0.072 | 0.007 |  | -0.048 | -0.142 | 0.046 | 0.320 |
| Apgar score (1 min) | 0.009 | -0.124 | 0.141 | 0.900 |  | 0.059 | -0.003 | 0.122 | 0.064 |
| Apgar score (5 min) | -0.082 | -0.293 | 0.130 | 0.449 |  | 0.088 | 0.008 | 0.169 | 0.032 |
| Variables at study* | |  |  |  |  |  |  |  |  |
| Postnatal age (day) | -0.020 | -0.032 | -0.007 | 0.002 |  | -0.008 | -0.014 | -0.002 | 0.009 |
| Post-conceptional age (week) | 0.087 | -0.082 | 0.256 | 0.311 |  | 0.047 | -0.043 | 0.137 | 0.304 |
| Body weight (kg) | 0.025 | -0.039 | 0.089 | 0.448 |  | -0.025 | -0.080 | 0.030 | 0.371 |
| Blood haemoglobin (g/dL) | 0.501 | 0.368 | 0.633 | <0.001 |  | 0.035 | -0.056 | 0.126 | 0.454 |
| Full enteral feeding ≥100mL/Kg/d (day) | -0.031 | -0.081 | 0.018 | 0.216 |  | -0.052 | -0.079 | -0.026 | <0.001 |
| $\boldsymbol{\mu}_{\boldsymbol{s}}^{\boldsymbol{'}}$(cm^-1^) | 0.581 | 0.344 | 0.818 | <0.001 |  | Not applicable | | | |
| µ_a_ (cm^-1^) | Not applicable | | | |  | 27.468 | 20.168 | 34.767 | <0.001 |

*Findings are adjusted for the position of the head.

**Assessed at 36 weeks post-conceptional age (or on day 28 for those born later than 32 weeks gestation).

Abbreviations: B, regression coefficient. CI, confidence interval. µ_a_, absorption coeffieicnt. $\boldsymbol{\mu}_{\boldsymbol{s}}^{\boldsymbol{'}}$, reduced scattering coefficient.

Online supplemental table 3: Dependence of µ_a_ and µ’_s_ obtained with the wavelength of 836nm on clinical variables: univariate analysis

|  | **Correlation with µ_a_·10^2^** | | | | | **Correlation with** $\boldsymbol{\mu}_{\boldsymbol{s}}^{\boldsymbol{'}}$ | | | |
| --- | --- | --- | --- | --- | --- | --- | --- | --- | --- |
| **Independent variables** | **B** | **95% CI** | | **P** |  | **B** | **95% CI** | | **P** |
|  |  | **Lower** | **Upper** |  |  |  | **Lower** | **Upper** |  |
| Position (vs. anterior) | | |  |  |  |  |  |  |  |
| Posterior | 3.172 | 2.822 | 3.521 | <0.001 |  | 1.312 | 1.041 | 1.583 | <0.001 |
| Right | 0.534 | 0.275 | 0.793 | <0.001 |  | 1.460 | 1.221 | 1.699 | <0.001 |
| Left | 0.249 | 0.027 | 0.470 | 0.025 |  | 0.849 | 0.620 | 1.078 | <0.001 |
| Maternal and antenatal variables* | | |  |  |  |  |  |  |  |
| Male sex | -0.488 | -1.156 | 0.180 | 0.152 |  | -0.165 | -0.471 | 0.141 | 0.290 |
| Multiple pregnancy | -0.763 | -1.324 | -0.202 | 0.008 |  | -0.256 | -0.629 | 0.116 | 0.177 |
| Antenatal glucocorticoid | -1.331 | -1.938 | -0.724 | <0.001 |  | -0.327 | -0.627 | -0.027 | 0.033 |
| Hypoglycaemia <48 h of birth | 0.945 | -1.298 | 3.188 | 0.409 |  | 0.632 | -0.201 | 1.464 | 0.137 |
| Variables at birth* | |  |  |  |  |  |  |  |  |
| Indomethacin for patent ductus arteriosus | -0.760 | -1.371 | -0.149 | 0.015 |  | -0.533 | -0.815 | -0.251 | <0.001 |
| Emergency caesarean delivery | -0.613 | -1.245 | 0.019 | 0.057 |  | -0.362 | -0.654 | -0.070 | 0.015 |
| Chronic lung disease** | -0.706 | -1.277 | -0.136 | 0.015 |  | -0.361 | -0.704 | -0.019 | 0.039 |
| Intraventricular haemorrhage | -0.480 | -0.937 | -0.023 | 0.039 |  | 0.198 | -0.492 | 0.888 | 0.574 |
| Gestational age (week) | 0.215 | 0.112 | 0.319 | <0.001 |  | 0.073 | 0.037 | 0.109 | <0.001 |
| Body weight (kg) | 0.157 | 0.100 | 0.213 | <0.001 |  | 0.037 | 0.013 | 0.060 | 0.002 |
| Z-score of above | 0.151 | -0.115 | 0.418 | 0.265 |  | -0.019 | -0.132 | 0.095 | 0.749 |
| Head circumference (cm) | 0.193 | 0.089 | 0.296 | <0.001 |  | 0.060 | 0.016 | 0.104 | 0.008 |
| Z-score of above | -0.033 | -0.442 | 0.376 | 0.875 |  | -0.122 | -0.261 | 0.018 | 0.088 |
| Cord blood pH per 0.1 change | -0.283 | -0.486 | -0.079 | 0.006 |  | -0.042 | -0.125 | 0.042 | 0.325 |
| Apgar score (1 min) | 0.052 | -0.089 | 0.194 | 0.471 |  | 0.073 | 0.015 | 0.131 | 0.014 |
| Apgar score (5 min) | -0.051 | -0.259 | 0.157 | 0.632 |  | 0.093 | 0.017 | 0.169 | 0.016 |
| Variables at study* | |  |  |  |  |  |  |  |  |
| Postnatal age (day) | -0.026 | -0.040 | -0.013 | <0.001 |  | -0.008 | -0.013 | -0.003 | 0.003 |
| Post-conceptional age (week) | 0.168 | -0.050 | 0.385 | 0.131 |  | 0.068 | -0.012 | 0.148 | 0.095 |
| Body weight (kg) | 0.078 | -0.014 | 0.170 | 0.095 |  | 0.002 | -0.036 | 0.040 | 0.919 |
| Blood haemoglobin (g/dL) | 0.619 | 0.498 | 0.739 | <0.001 |  | 0.057 | -0.015 | 0.128 | 0.119 |
| Full enteral feeding ≥100mL/Kg/d (day) | -0.052 | -0.103 | 0.000 | 0.048 |  | -0.051 | -0.075 | -0.026 | <0.001 |
| $\boldsymbol{\mu}_{\boldsymbol{s}}^{\boldsymbol{'}}$(cm^-1^) | 0.653 | 0.345 | 0.961 | <0.001 |  | Not applicable | | | |
| µ_a_ (cm^-1^) | Not applicable | | | |  | 20.742 | 14.989 | 26.496 | <0.001 |

*Findings are adjusted for the position of the head.

**Assessed at 36 weeks post-conceptional age (or on day 28 for those born later than 32 weeks gestation).

Abbreviations: B, regression coefficient. CI, confidence interval. µ_a_, absorption coeffieicnt. $\boldsymbol{\mu}_{\boldsymbol{s}}^{\boldsymbol{'}}$, reduced scattering coefficient.

Online supplemental table 4: Dependence of µ_a_ and µ’_s_ obtained with the wavelength of 761nm on clinical variables: multivariate analysis

|  | **Correlation with µ_a_·10^2^** | | | | | **Correlation with** $\boldsymbol{\mu}_{\boldsymbol{s}}^{\boldsymbol{'}}$ | | | |
| --- | --- | --- | --- | --- | --- | --- | --- | --- | --- |
|  | **B** | **95% CI** | | **P** |  | **B** | **95% CI** | | **P** |
|  |  | **Lower** | **Upper** |  |  |  | **Lower** | **Upper** |  |
| Independent variables |  |  |  |  |  |  |  |  |  |
| Body weight at birth (per 100g) | -0.020 | -0.068 | 0.028 | 0.417 |  | -0.010 | -0.039 | 0.019 | 0.509 |
| Apgar score (5 min) | -0.035 | -0.224 | 0.154 | 0.715 |  | 0.096 | 0.028 | 0.165 | 0.006 |
| Full enteral feeding ≥100mL/Kg/d (day) | 0.049 | 0.014 | 0.084 | 0.006 |  | -0.052 | -0.081 | -0.024 | <0.001 |
| Post-conceptional age at study (week) | 0.145 | 0.028 | 0.261 | 0.015 |  | 0.072 | 0.006 | 0.137 | 0.033 |
| Covariates |  |  |  |  |  |  |  |  |  |
| Antenatal glucocorticoid | -0.244 | -0.663 | 0.176 | 0.255 |  | -0.058 | -0.369 | 0.253 | 0.715 |
| Multiple pregnancy | -0.181 | -0.507 | 0.146 | 0.278 |  | -0.171 | -0.518 | 0.175 | 0.333 |
| Male sex | -0.044 | -0.443 | 0.355 | 0.830 |  | -0.237 | -0.522 | 0.048 | 0.103 |
| Blood haemoglobin (g/dL) | 0.591 | 0.473 | 0.710 | <0.001 |  | Not involved | | | |
| $\boldsymbol{\mu}_{\boldsymbol{s}}^{\boldsymbol{'}}$(cm^-1^) | 0.354 | 0.230 | 0.478 | <0.001 |  | Not applicable | | | |
| µ_a_ (cm^-1^) | Not applicable | | | |  | 19.716 | 11.984 | 27.448 | <0.001 |

The model is also adjusted for the position of the head.

Abbreviations: B, regression coefficient. CI, confidence interval. µ_a_, absorption coeffieicnt. $\boldsymbol{\mu}_{\boldsymbol{s}}^{\boldsymbol{'}}$, reduced scattering coefficient.

Online supplemental table 5: Dependence of µ_a_ and µ’_s_ obtained with the wavelength of 791nm on clinical variables: multivariate analysis

|  | **Correlation with µ_a_·10^2^** | | | | | **Correlation with** $\boldsymbol{\mu}_{\boldsymbol{s}}^{\boldsymbol{'}}$ | | | |
| --- | --- | --- | --- | --- | --- | --- | --- | --- | --- |
|  | **B** | **95% CI** | | **P** |  | **B** | **95% CI** | | **P** |
|  |  | **Lower** | **Upper** |  |  |  | **Lower** | **Upper** |  |
| Independent variables |  |  |  |  |  |  |  |  |  |
| Body weight at birth (per 100g) | -0.036 | -0.087 | 0.015 | 0.163 |  | -0.034 | -0.069 | 0.001 | 0.056 |
| Apgar score (5 min) | -0.013 | -0.156 | 0.130 | 0.858 |  | 0.099 | 0.035 | 0.163 | 0.002 |
| Full enteral feeding ≥100mL/Kg/d (day) | 0.013 | -0.016 | 0.041 | 0.379 |  | -0.055 | -0.083 | -0.028 | <0.001 |
| Post-conceptional age at study (week) | 0.105 | -0.009 | 0.219 | 0.072 |  | 0.044 | -0.027 | 0.115 | 0.221 |
| Covariates |  |  |  |  |  |  |  |  |  |
| Antenatal glucocorticoid | -0.224 | -0.599 | 0.151 | 0.241 |  | -0.053 | -0.364 | 0.258 | 0.737 |
| Multiple pregnancy | -0.209 | -0.537 | 0.119 | 0.212 |  | -0.119 | -0.461 | 0.223 | 0.494 |
| Male sex | -0.148 | -0.508 | 0.212 | 0.419 |  | -0.236 | -0.516 | 0.043 | 0.097 |
| Blood haemoglobin (g/dL) | 0.529 | 0.424 | 0.634 | <0.001 |  | Not involved | | | |
| $\boldsymbol{\mu}_{\boldsymbol{s}}^{\boldsymbol{'}}$(cm^-1^) | 0.513 | 0.335 | 0.691 | <0.001 |  | Not applicable | | | |
| µ_a_ (cm^-1^) | Not applicable | | | |  | 28.516 | 20.240 | 36.792 | <0.001 |

The model is also adjusted for the position of the head.

Abbreviations: B, regression coefficient. CI, confidence interval. µ_a_, absorption coeffieicnt. $\boldsymbol{\mu}_{\boldsymbol{s}}^{\boldsymbol{'}}$, reduced scattering coefficient.

Online supplemental table 6: Dependence of µ_a_ and µ’_s_ obtained with the wavelength of 836nm on clinical variables: multivariate analysis

|  | **Correlation with µ_a_·10^2^** | | | | | **Correlation with** $\boldsymbol{\mu}_{\boldsymbol{s}}^{\boldsymbol{'}}$ | | | |
| --- | --- | --- | --- | --- | --- | --- | --- | --- | --- |
|  | **B** | **95% CI** | | **P** |  | **B** | **95% CI** | | **P** |
|  |  | **Lower** | **Upper** |  |  |  | **Lower** | **Upper** |  |
| Independent variables |  |  |  |  |  |  |  |  |  |
| Body weight at birth (per 100g) | 0.011 | -0.033 | 0.056 | 0.617 |  | -0.018 | -0.042 | 0.006 | 0.148 |
| Apgar score (5 min) | 0.016 | -0.128 | 0.160 | 0.825 |  | 0.091 | 0.029 | 0.154 | 0.004 |
| Full enteral feeding ≥100mL/Kg/d (day) | 0.019 | -0.011 | 0.048 | 0.218 |  | -0.045 | -0.069 | -0.021 | <0.001 |
| Post-conceptional age at study (week) | 0.178 | 0.044 | 0.313 | 0.009 |  | 0.052 | -0.008 | 0.111 | 0.088 |
| Covariates |  |  |  |  |  |  |  |  |  |
| Antenatal glucocorticoid | 0.003 | -0.402 | 0.408 | 0.988 |  | 0.018 | -0.265 | 0.300 | 0.903 |
| Multiple pregnancy | -0.373 | -0.761 | 0.015 | 0.060 |  | -0.167 | -0.484 | 0.149 | 0.301 |
| Male sex | -0.043 | -0.413 | 0.327 | 0.819 |  | -0.190 | -0.446 | 0.066 | 0.145 |
| Blood haemoglobin (g/dL) | 0.587 | 0.477 | 0.698 | 0.988 |  | Not involved | | | |
| $\boldsymbol{\mu}_{\boldsymbol{s}}^{\boldsymbol{'}}$(cm^-1^) | 0.469 | 0.319 | 0.619 | <0.001 |  | Not applicable | | | |
| µ_a_ (cm^-1^) | Not applicable | | | |  | 20.489 | 14.292 | 26.685 | <0.001 |

The model is also adjusted for the position of the head.

Abbreviations: B, regression coefficient. CI, confidence interval. µ_a_, absorption coeffieicnt. $\boldsymbol{\mu}_{\boldsymbol{s}}^{\boldsymbol{'}}$, reduced scattering coefficient.
